# Loss of ASMT Function in Arabidopsis Affects Hormone Pathways and the Ability to Withstand Drought Stress

**DOI:** 10.3390/ijms27135737

**Published:** 2026-06-25

**Authors:** Victoria V. Shitikova, Ivan A. Bychkov, Anna V. Klepikova, Anna S. Lifanova, Natalia V. Kudryakova, Elena S. Pojidaeva, Victor V. Kusnetsov

**Affiliations:** 1 K.A. Timiryazev Institute of Plant Physiology, Russian Academy of Sciences, Moscow 119991, Russia; vicry@yandex.ru (V.V.S.); ivan.a.b@mail.ru (I.A.B.); ana.unw7@yandex.ru (A.S.L.); alenapoj@mail.ru (E.S.P.); vkusnetsov2001@mail.ru (V.V.K.); 2 N.I. Vavilov Institute of General Genetics, Russian Academy of Sciences, Moscow 119991, Russia; annklepikova@gmail.com

**Keywords:** *Arabidopsis thaliana*, dehydration stress, melatonin, *N*-acetylserotonin methyltransferase, phytohormones

## Abstract

*N*-acetylserotonin methyltransferase (ASMT) is among the key enzymes involved in the final steps of melatonin biosynthesis. Here, we have shown that inactivation of *ASMT* in *A. thaliana* results in reduced endogenous melatonin levels, modulating other plant hormone pathways and affecting stress-related responses. Transcriptomic analysis of the asmt-null mutant revealed that the differentially expressed genes were predominantly enriched in terms associated with auxin responses and signalling, as well as with abscisic acid (ABA)-mediated stress responses. In addition, the expression of genes involved in the ethylene, salicylic acid, jasmonic acid and brassinosteroid pathways was altered in the mutant. Assays of a β-glucuronidase (GUS) construct in which a fragment containing 1000 bp upstream of the *ASMT* start codon was fused to the *GUS* reporter gene confirmed that ASMT is involved in the responses to ABA, gibberellic and indole acetic acids, *trans*-zeatin, ethylene and epibrassinolide, which is consistent with the results of the in silico analysis of the *ASMT* promoter. Furthermore, the expression of a number of genes, such as *SLG1*, *HIS1-3*, *AtAIRP1* and several *LEA* genes, whose transcriptional regulation is associated with water management and contributes to impaired tolerance to dehydration stress, was altered in the mutant. The pleiotropic effects of *ASMT* gene disruption facilitate the identification of new potential melatonin targets and provide insights into the specific mechanisms of melatonin action.

## 1. Introduction

The irrevocable step in melatonin (MT) biosynthesis is catalyzed by *N*-acetylserotonin methyltransferase (ASMT). In the presence of intermediates, the enzyme either converts *N*-acetylserotonin into MT or catalyzes the conversion of serotonin into *N*-acetylserotonin, which is then converted into MT by serotonin *N*-acetyltransferase (SNAT) [1,2,3]. ASMT is involved in the terminal reaction in the cytoplasm, whereas SNAT is believed to be the final step enzyme in chloroplasts. At low cellular *N*-acetylserotonin levels, ASMT enzymes preferentially convert *N*-acetylserotonin into MT, whereas at high cellular serotonin levels, ASMT converts serotonin to 5-methoxytryptamine, which is then converted into MT by SNAT [1]. Similar steps of MT synthesis can also be catalyzed by COMT (caffeic acid O-methyltransferase), which functions similarly to ASMT and plays a key role in the final steps of plant MT synthesis.

*ASMT* transcripts are rapidly induced under stress conditions, which is consistent with their function in the defence response against various adverse environments. This induction occurs in parallel with the induction of *TDC* (tryptophan decarboxylase) and *T5H* (tryptamine 5-hydroxylase), which are involved in the prior steps of MT synthesis. In contrast, the expression of *SNAT1* and *SNAT2* is downregulated [1].

To date, a number of *ASMT* genes have been described in more than 10 species, including the *ASMT1–3* gene family in rice, which shares significant sequence identity, in addition to the rice *ASMT5*, which does not show significant sequence homology with *ASMT1–3* [2]. Forting genes encoding ASMT proteins have been identified in the tomato genome, 7 of which exhibit broad expression across various plant tissues [4]. Five *ASMT* genes have been revealed in the *Hypericum perforatum* genome [5], and at least 16 *CaASMT* genes have been identified in pepper [6]. Furthermore, a total of 37 *ASMT* genes were identified in apple, two of which, *MdASMT11* and *MdASMT14*, were strongly upregulated by heat, cold, drought, and NaCl stress [7]. Genome-wide identification of the walnut *N*-acetylserotonin methyltransferase gene family by means of bioinformatics analysis revealed 46 putative *ASMT* genes distributed in clusters on 10 chromosomes, 4 of which were homologous both within walnut and between species [8]. A total of 20 *ASMT* genes distributed on seven chromosomes have been identified in tea plants [9].

According to computer predictions, the genes in the *ASMT* gene family may include the greatest number of members among the MT biosynthesis genes, with 19 and 28 *ASMT* genes present in rice and sorghum, respectively, and 17 and 11 *ASMT* genes in *Arabidopsis* and tomato, respectively [10]. The multiple putative members identified in the ASMT family branched into two major clusters in the phylogenetic tree, one constituting ASMTs from rice and sorghum and the other comprising *Arabidopsis* and tomato proteins. However, as the authors admit, the selection criteria for AtASMT proteins were relaxed for protein sequences with a percent identity of 30%, since no hits were found at the identity threshold of 40%. Hence, further research is needed to determine the precise role of the predicted AtASMT proteins in MT synthesis.

In *Arabidopsis*, only the recombinant protein expressed from the *At4g35160* gene has been shown to possess ASMT enzyme activity [11]. The AtASMT protein is localized in the cytoplasm and catalyzes the conversion of N-acetylserotonin to MT and serotonin to 5-methoxytryptamine, with Vmax values of 0.11 and 0.29 pcat/mg protein, respectively. Its catalytic efficiency (*K*cat/km) toward serotonin was comparable to that of *N*-acetylserotonin, indicating that ASMT enzymes can accept both substrates with similar catalytic efficiency. However, the low catalytic efficiency of ASMT and SNAT, which are involved in the last steps of MT biosynthesis, suggests that MT biosynthesis is strictly regulated. Furthermore, MT can be rapidly metabolized into 2-hydroxyMT (2-OHMel) and cyclic3-hydroxyMT (3-OHMel) by MT 2-hydroxylase (M2H) and MT 3-hydroxylase (M3H), respectively [2], which maintain the equilibrium level required for both defence responses and function as signalling molecules during plant growth and development.

According to the results of the Affymetrix analysis (https://bar.utoronto.ca/efp/cgi-bin/efpWeb.cgi, accessed on 21 May 2026), the expression profile of ASMT was organ dependent, with the highest levels in green embryos of freshly harvested seeds and in the floral meristem. When dry seeds were stored at 22 °C, the relative abundance of *ASMT* transcripts gradually decreased. During stratification in the dark at 4 °C, the relative expression level of the *ASMT* decreased until it completely disappeared after 48 h. The expression of the ASMT in the stratified seeds increased threefold after 48 h. After 48 h, the ASMT expression level varied within low values in rosette leaves, shoot tips and mature siliques, whereas in floral tissue, the relative transcript level increased. These findings indicate that the functional activity of *ASMTs* under normal conditions may be associated with various stages of plant growth and development.

Different levels of MT and its metabolites can cause diverse physiological effects when MT is either overproduced or applied exogenously. Overexpression of *AtASMT* caused massive MT accumulation and synergized with the phytohormone abscisic acid (ABA) to inhibit seed germination in *Arabidopsis* [12]. On the other hand, ABA consistently suppressed *AtASMT* under high light stress despite the differential response of other MT biosynthesis genes [13]. These findings suggest that plants exhibit context-dependent MT responses defined by their genetic background and physiological status. Therefore, effective strategies for MT application, whether through the generation of transgenic crops or exogenous treatment, require in-depth studies of reliable MT target genes and the mechanism by which they participate in the fine-tuning of MT-responsive physiological processes.

The aim of this work was to define a set of MT-affected genes and their role in biological processes by using a reverse genetics approach. Using the results of RNA-Seq analysis of an *Arabidopsis* mutant with a disrupted *ASMT* gene and reduced endogenous levels of MT, we initiated a functional characterization of MT targets in MT-sensitive processes. The *asmt* mutant exhibited altered expression of a set of genes associated mainly with hormone regulation and stress responses. We found that the activity of the *ASMT* promoter is driven by abscisic acid (ABA), gibberellic acid, indole acetic acid, *trans*-zeatin (tZ), ethylene and epibrassinolide (EBL) treatment. Furthermore, disruption of *ASMT1* increases ROS production and augments drought sensitivity because of the impaired function of draught-responsive genes.

## 2. Results

### 2.1. Phenotype of the asmt-KO Arabidopsis Mutant

The *AtASMT* gene (*AT4G35160*) is 2221 bp long with 2 exons and 1 intron and is located on chromosome 4. The *asmt*-knockout mutant line chosen for this study (NASC680911) had T-DNA inserted in the intron between the first and second exons. After screening the homozygous line, the expression levels of *ASMT* were determined. The mutant exhibited no *ASMT* transcripts (Figure S1).

**Figure 1:**
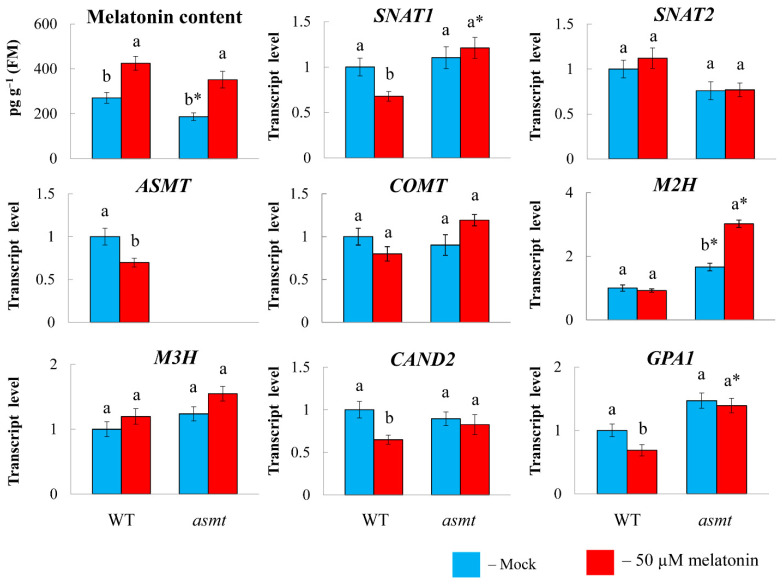
MT content and expression of genes involved in MT synthesis, catabolism, and signalling in wild type and mutant *asmt*. The data presented are the mean values ± SEs (*n* ≥ 3). Different letters denote statistically significant differences at *p* < 0.05 between the control and treatment (*t* test). Asterisks indicate statistically significant differences at *p* < 0.05 between the wild type and mutant. (*t* test).

Under standard growth conditions (100 μmol·m^−2^·s^−1^, 23 °C, 16 h photoperiod), the *asmt* mutant displayed a morphological phenotype similar to that of the wild type (WT). However, the loss of ASMT function resulted in a significant decrease in the MT content in two-week-old rosette leaves, as well as unchanged expression of the MT synthesis genes *SNAT1* and *COMT* and a slight, although not statistically significant, decrease in *SNAT2* expression, whereas the transcript levels of the metabolism gene *M2H* increased, as determined by qRT-PCR analysis (Figure 1). We also validated whether the transcript levels of these genes were feedback-regulated by MT. MT treatment (50 μM, 24 h) caused a significant decrease in *ASMT* and *SNAT1* expression but did not substantially affect the accumulation of *SNAT2, M2H* and *M3H* transcripts in the wild type. Furthermore, the transcript levels of the putative MT signalling pathway gene *CAND2* and its associated G protein-encoding gene *GPA1* were also significantly reduced in MT-treated wild type plants, which is consistent with a feedback mechanism. However, these genes did not respond to MT in *asmt*, except for *M2H* (Figure 1).

To explore directly whether reduced MT levels in *asmt* plants affect antioxidant activity, the total antioxidant capacity, hydrogen peroxide level and activity of antioxidant enzymes were investigated. As expected, compared with the wild type, the mutant exhibited slightly reduced total antioxidant capacity, which increased more substantially after MT treatment (Table 1). The elevated levels of hydrogen peroxide in the mutant were combined with increased levels of peroxidase (POD) and superoxide dismutase (SOD) activity, which are directly involved in scavenging mechanisms. Moreover, the activity of these enzymes decreased under the influence of MT only in the *asmt*, indicating its increased sensitivity to the regulator.

As a consequence of the misregulation of MT synthesis, the mutant also displayed a 20% decrease in control proline levels, suggesting reduced osmoprotective and ROS scavenging capacity. However, no significant difference in the levels of thiobarbituric acid reactive substances (TBARs) or electrolyte leakage was detected, indicating that the membrane cell wall integrity of the *asmt* mutant was not impaired under control conditions. Taken together, these data provide direct evidence that impaired *ASMT* is functionally coupled with increased ROS production.

### 2.2. Asmt Mutation Alters the Expression of Hormone- and Stress-Responsive Genes

**Figure 2:**
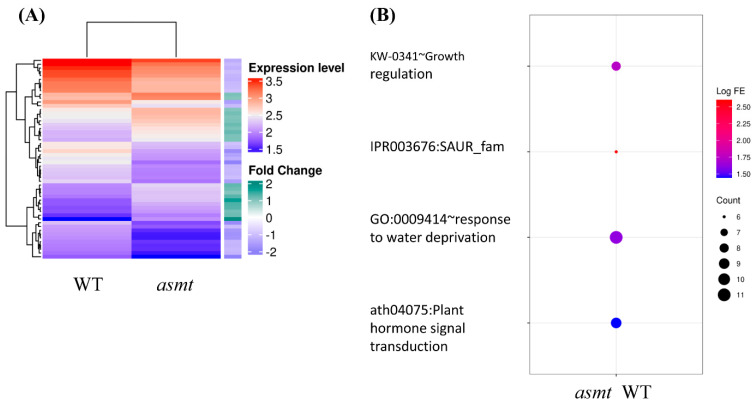
The expression profiles and functional enrichment of DEGs in Col-0 and *asmt* mutant. (**A**) Transcriptome heatmap showing the expression patterns of DEGs affected by the *asmt* mutation under normal conditions. The color of the heatmap represents the logarithm of the mean expression level in the sample. The color of the annotation represents the logarithm of the fold change in gene expression between the *asmt* mutant and the wild type. (**B**) Term enrichment of the genes significantly differentially expressed in the *asmt* mutant under normal conditions determined by the Gene Ontology (GO), KEGG and InterPro databases. The logFEs indicated by the color represent the logarithm of the fold enrichment values, and the size of the dots represents the number of DEGs annotated with the term.

To identify a potential link between the genetic inactivation of the *AtASMT* gene and molecular effects, we performed transcriptomic profiling of 2-week-old mutant plants. Genes whose |log2(fold change)| was ≥ 1.0 and whose adjusted *p* value was < 0.05 were considered significantly differentially expressed. MT deficiency in the *asmt* mutant induced the upregulation of 22 differentially expressed genes (DEGs) and the downregulation of 43 DEGs (|log2 (fold change)| ≥ 1, adjusted *p* value < 0.05) compared with those in the control Col-0 plants (Figure 2). To query presumable physiological processes potentially targeted by these transcriptional changes, we analysed the enrichment of the DEGs identified by RNA-seq. GOTERM_BP_DIRECT classification of DEGs into biological functions revealed enriched GO terms associated with water deficiency (GO:0009414). The DEGs present in the GO-enriched group included several genes encoding members of the late embryogenesis abundant (LEA) protein family, which are associated with tolerance to freezing and desiccation. One of them, *ABR* (*AT3G02480*), which belongs to the LEA 4 family, also delays dark-induced leaf senescence and is negatively regulated by ABI5 [14]. Among the genes associated with water deficiency are *SLG1* (*AT5G08490*), encoding a mitochondrion-localized PPR protein involved in RNA editing [15], and *AtAIRP* (*AT4G23450*), which acts as a positive regulator of ABA-dependent stress responses [16]. Another gene, *HIS1-3* (*AT2G18050*), encodes a structurally distinct linker histone that functions in chromatin folding. *HIS1-3* mediates adaptive responses to complex environmental stressors by facilitating chromatin accessibility and is highly dependent on ABA [17].

In addition, the *asmt* mutant has reduced expression of the *MYB76* (*AT5G07700*) gene, encoding a transcription factor for the biosynthesis of aliphatic glucosinolates, which may contribute to a decrease in the concentration of 4-methoxyindol-3-ylmethyl glucosinolate and an increased vulnerability to phloem-feeding insects, especially under drought conditions [18].

In summary, most of the aforementioned genes are involved in ABA-mediated stress responses.

As a complimentary approach to the GO term enrichment analysis, we applied KEGG pathway classification according to which the DEGs were enriched in terms of plant hormone signal transduction (KEGG_PATHWAY: ath04075). These genes belong mainly to auxin metabolic and signalling pathways, which are involved in a wide range of biological processes (*IAA6* and several *SAUR* family genes). The functions of highly repressed *HAI2* (*AT1G07430*) in this group are associated with drought-associated signalling as well as negative regulation of the abscisic acid-activated signalling pathway and positive regulation of gibberellic acid-mediated signalling [19,20], whereas *PR1* (*AT2G14610*) plays a role in multiple stress responses and is induced by salicylic acid [21]). In addition, the expression of genes involved in ethylene, SA, jasmonic acid (Ja) and brassinosteroid responses was altered in the mutant. More detailed annotations of the DEGs in the *asmt* mutant compared to the wild type are provided in Supplementary Table S1. In total, the *asmt* mutation resulted in a high proportion of DEGs affecting hormone metabolic and signalling pathways, suggesting that MT deficiency modulates almost all classes of plant hormones.

### 2.3. Identification of Cis-Regulatory Elements in the ASMT Promoter

To gain insight into ASMT regulatory networks in response to different types of stimuli, the *ASMT* promoter was analysed. According to the PlantProm DB database (https://ppdb.agr.gifu-u.ac.jp/ppdb/cgi-bin/index.cgi, accessed on 15 November 2024), the basal promoter of the *ASMT* gene consists of 1000 bp, while its maximum length can reach 2483 bp as per the AGRIS database (https://agris-knowledgebase.org/AtcisDB/getpromseq.html?id=At4g35160, accessed on 15 November 2024).

Detailed in silico analysis of the selected gene region revealed that the promoter contains *cis*-acting elements recognized by transcription factors from various regulatory families. They participate in the transcriptional regulation of biological processes associated with both plant growth and development (embryo development and seed germination, floral meristem formation, lateral root formation, and the transition to senescence) and responses to biotic and abiotic stimuli (pathogen invasion, temperature changes, light parameters, and hypoosmolarity). General information on the *cis*-elements and *trans*-factor (TF) families obtained using AGRIS is presented in Table S2.

To date, extensive efforts to experimentally define MT-driven binding sites shared by MT-responsive genes, as well as the elements of the MT signal transduction pathway, have proven unsuccessful despite the discovery of the MT receptor in plants. However, the cross-interaction of MT with all known classes of phytohormones implies that MT may act in concert with hormone transcription factors. Putative hormone-responsive *cis*-elements in the *ASMT* promoter were linked to the responses to ethylene, jasmonic acid, brassinosteroids, ABA, cytokinin (CK), and auxin. Therefore, hormone-mediated regulation of *ASMT* may contribute to the modulation of endogenous MT levels, thereby attaining specificity to MT effects.

### 2.4. Plant Hormones Regulate the Expression of ASMT and GUS Activity in the ASMT::GUS Transgenic Line

To test whether the results of the *ASMT* promoter analysis are consistent with those of the *ASMT* promoter activity in response to hormone treatment and to further identify potential hormone-driven motifs in the *ASMT* promoter, we generated a transgenic line in which a fragment 1000 bp upstream of the *ASMT* start codon was fused to the open reading frame of the β-glucuronidase (GUS) reporter gene. The size of the deletion fragment was nearly identical to the size of the basal promoter of the *ASMT* gene.

Seven-day-old *Arabidopsis* seedlings were selected for the analysis of β-glucuronidase activity, as the strongest *ASMT* promoter-driven GUS activity was observed in young seedlings. At later stages, GUS activity generally decreased.

**Figure 3:**
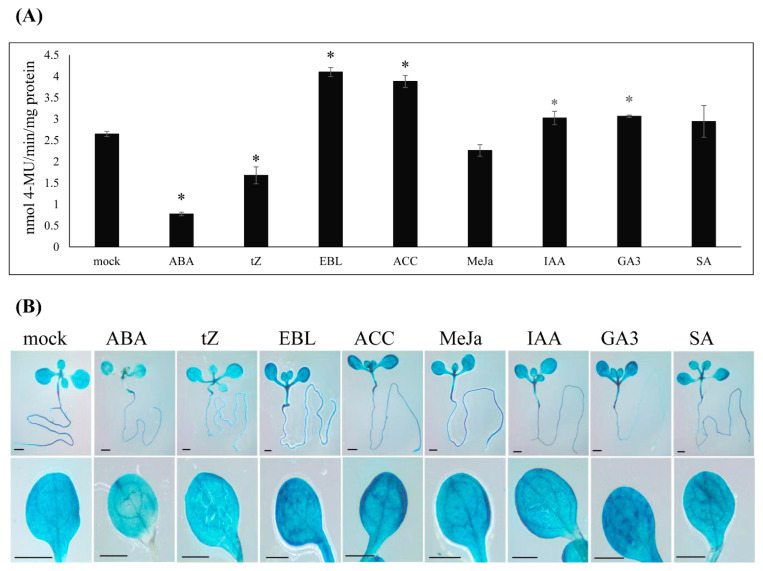
*ASMT* promoter activity in *A. thaliana* plants. (**A**) Fluorometric assay of GUS activity measured with 4-MUG of the *ASMT* promoter (P1000) in *Arabidopsis* plants after 24 h of exposure to the appropriate phytohormone or without it (mock). The bars indicate the means ± SDs of 3 biological replicates. Asterisks indicate statistically significant differences at *p* < 0.05 between the hormone-treated and untreated (mock) plants (*t* test). (**B**) Histochemical staining of GUS activity measured with x-Gluc of the *ASMT* promoter (P1000) in plants after 24 h of exposure to the appropriate phytohormone or without it (mock). Large-scale = 1 mm.

Evaluation of the GUS activity in transgenic plants containing P1000 by the fluorometric method displayed that it was activated by 1-aminocyclopropane-1-carboxylic acid (ACC), EBR, indole acetic acid (IAA) and gibberellic acid (GA3), slightly repressed by methyl jasmonate (MeJa) but remained practically unchanged when the seedlings were treated with salicylic acid (SA) (Figure 3A). At the same time ABA, and tZ treatment significantly repressed it. These results were also confirmed by x-Gluc staining images (Figure 3B). Thus, the analysis of the expression of the *GUS* marker gene under the control of the P1000 fragment allows us to conclude that *ASMT* and its product are involved in responses to ABA, GA3, IAA, tZ, ethylene and EBS, in accord with the results of in silico promoter analysis.

Histochemical staining of GUS activity measured with x-Gluc of the *ASMT* promoter (P1000) in plants after 24 h of exposure to the appropriate phytohormone or without it (mock). Large-scale = 1 mm. (B) Fluorometric assay of GUS activity measured with 4-MUG of the *ASMT* promoter (P1000) in *Arabidopsis* plants after 24 h of exposure to the appropriate phytohormone or without it (mock). The bars indicate the means ± SDs of 3 biological replicates. Asterisks indicate statistically significant differences at *p* < 0.05 between the hormone-treated and untreated (mock) plants (*t* test).

**Figure 4:**
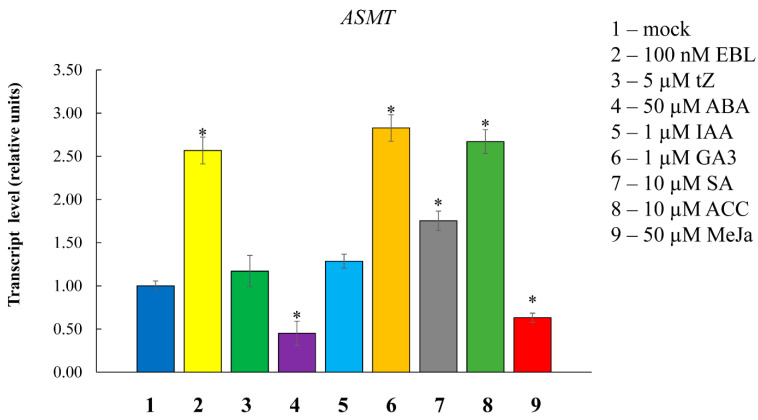
*ASMT* gene expression (qRT-PCR) in the wild type without treatment (mock) and with different hormone treatments. The data presented are the mean values ± SEs (*n* ≥ 3). Asterisks indicate statistically significant differences at *p* < 0.05 between hormone-treated and untreated (mock) plants (*t* test).

To further determine whether *ASMT* expression is altered in a hormone-dependent manner, we assessed *ASMT* transcript accumulation in response to phytohormones. Treatment of 7-day-old WT seedlings with a set of 8 classical hormones resulted in more than twofold upregulation of *ASMT* expression by EBL, GA3, and ACC and downregulation by MeJa and ABA, whereas transcript levels did not change significantly after treatment with IAA, tZ or SA, despite the response of the corresponding hormone marker genes indicating the effectiveness of hormone application (Figure 4, Table S3). Overall, these results indicate that *ASMT* expression is hormone responsive.

### 2.5. Modified Responses of the Asmt Mutant to Hormone Treatment

**Figure 5:**
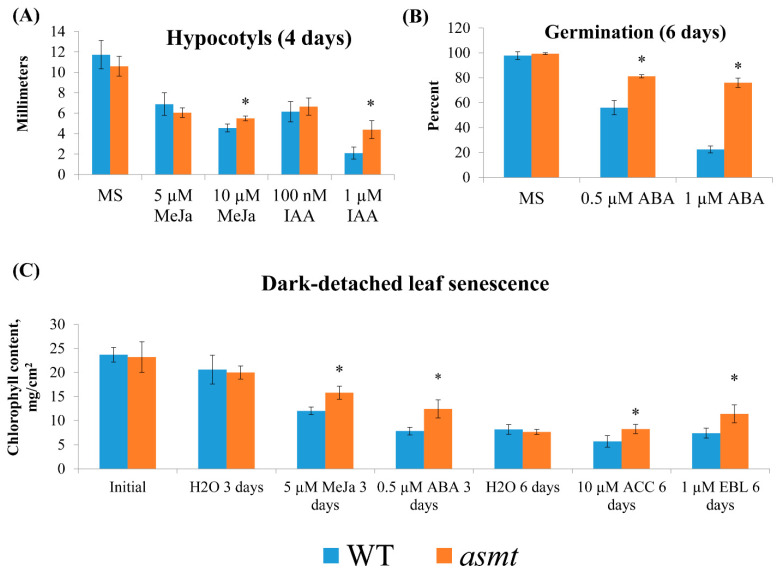
Hypocotyl length (**A**), seed germination quality (**B**) and leaf senescence rate (**C**) in the wild type and the *asmt* mutant without treatment and with hormone treatment. The data presented are the mean values ± SEs (*n* ≥ 3). Asterisks indicate statistically significant differences at *p* < 0.05 between the wild type and mutant. (*t* test).

On the basis of the results of the transcriptomic and *ASMT* promoter analyses, we next asked whether disruption of *ASMT* alters physiological responses to hormone treatment through standard sensitivity tests. In the hypocotyl growth assay, the mutant displayed decreased sensitivity to IAA (1 µM) and MeJa (10 µM) (Figure 5A). In parallel, compared with the WT plants, the mutant plants reached markedly greater levels of seedling establishment in response to different concentrations of ABA during seed germination (Figure 5B) and were also less sensitive to ABA in the dark-detached leaf assay (Figure 5C). Furthermore, the mutant exhibited reduced sensitivity to ACC and MeJA in terms of promoting leaf senescence in detached leaves but was more sensitive to EBL in terms of the retardation of chlorophyll loss. Overall, these results demonstrate that the altered sensitivity of ASMT to hormonal stimuli is dependent on temporal and tissue-specific effects.

We further asked whether hormone-related genes whose basal transcription levels are altered in the mutant might be regulated differently by relevant hormones than they are in the wild type. To this end, *asmt* plants were subjected to hormone treatment, and the transcriptional responses of the selected genes were compared with those of WT plants. Quantitative RT-PCR analysis revealed diverse responses of various genes to hormone treatment. The expression patterns of some transcripts that were either up- or downregulated in the WT were similar to those in *asmt*, although to a greater or lesser extent (e.g., *NCED3* by ABA, *PR1* by SA, *ACS8* by ACC, and *RAP2.6* by ABA or MeJa) (Table 2). Specifically, compared with the wild-type plants, the plants in which PR1 expression decreased to basal levels in *asmt* were approximately 4-fold lower in response to SA treatment. This response implies that the mutant has defects in the regulation of adaptive responses against both biotic and abiotic stresses, given that *PR1* is induced by a variety of pathogens that activate immune reactions [21] and is also involved in the response to water deprivation [22,23].

On the other hand, the hormone-mediated repression of certain hormone signalling genes in the wild type was reversed (e.g., *ERF14* by ACC, *HAI1* by GA3 or *SAUR21* by IAA). The opposite pattern of regulation can be considered as part of the diverse adaptive mechanisms that may operate with a limited endogenous MT content.

### 2.6. Physiological and Transcriptional Responses to Drought Are Impaired in the asmt Mutant

Since the differentially expressed genes in the *asmt* mutant were enriched in GO terms related to ABA-mediated stress responses, particularly water deficit, we also investigated whether *ASMT* disruption contributes to altered responses to stress conditions. To address this concern, two types of experiments were performed. We used qRT-PCR to assess the transcript abundance of the selected drought-responsive genes. As a complimentary approach, the sensitivity of the mutant to drought stress was examined.

**Figure 6:**
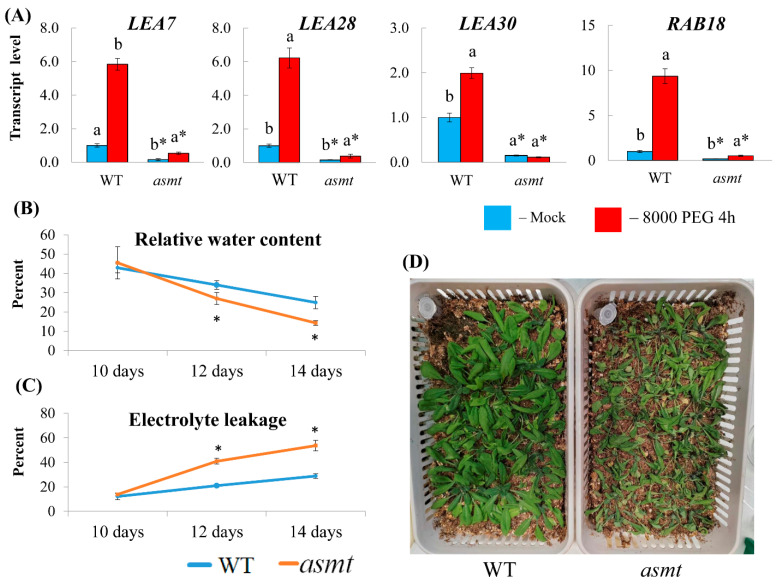
(**A**) Expression of genes (qRT-PCR) of the *LEA* family and the *RAB18* gene (**A**), in the wild type and the *asmt* mutant under water deficit imposed by PEG treatment. (**B**) Relative water content and (**C**) electrolyte yield in the soil-grown wild type and the *asmt* mutant subjected to long-term drought treatment. The data presented are the mean values ± SEs (*n* ≥ 3). Different letters denote statistically significant differences at *p* < 0.05 between the control and treatment groups (ANOVA with post hoc Tukey’s multiple-comparison test or *t* test). Asterisks indicate statistically significant differences at *p* < 0.05 between the wild type and mutant. (*t* test). (**D**) Images of three-week-old soil-grown plants (wild type and *asmt* mutant) subjected to prolonged drought.

Validation of the transcriptomic data by qRT-PCR revealed altered steady-state expression levels of DEGs encoding late embryogenesis abundant (LEA) proteins and *RAB18*, encoding a member of the dehydrin family of proteins, that can protect other proteins from damage through binding to anionic phospholipids (Figure 6A) [24].

The transcript abundance of selected DEGs was further evaluated in response to water deficit imposed by PEG treatment. To this end, 2-week-old seedlings were incubated in liquid Murashige and Skoog (MS) media supplemented with 25% (*w*/*v*) polyethylene glycol 8000 (PEG) for 4 h [25]. The increase in the transcript levels of some *LEA* genes in response to PEG treatment in the *asmt* mutant plants was weaker than that in the wild type plants (Figure 6). These findings suggest that ASMT deficiency contributes to increased sensitivity to water deprivation by modulating the expression of target genes involved in drought tolerance.

To analyse the consequences of water deficit in the context of whole-plant physiology, the tolerance of 3-week-old WT and mutant plants to long-term drought treatment was tested on dry soil as described previously [26]. The significantly lower number of *asmt* plants that survived dehydration stress, as well as their lower relative water content, indicate that loss of function of *asmt* mutation decreases drought tolerance (Figure 6B,D). Compared with the WT plants, the mutant plants also presented significantly greater levels of ion leakage, suggesting that membrane integrity is reduced in the *asmt* under stress conditions (Figure 6C). Hence, the increased sensitivity of the mutant to dehydration stress corresponded to an alteration in its transcriptomic response.

## 3. Discussion

MT, which is considered a newly characterized phytohormone, plays a vital role in abiotic stress tolerance. However, the mechanisms underlying the effects of MT on stress responses in terms of crosstalk with phytohormones are still unclear. The beneficial role of MT has been proven predominantly in experiments involving exogenous supplementation of MT. However, one potential complication of such an experimental design is that some MT responses may be near saturation, particularly in wild type plants. In this work, using reverse genetics, we attempted to identify MT targets by analysing an *asmt Arabidopsis* mutant with a knockout of the MT synthesis gene.

The enzyme encoded by the *ASMT* gene is involved in the terminal steps of melatonin biosynthesis. It is closely coupled to regulation of the levels of melatonin and its metabolites which act as biostimulant or signaling molecules for a serious of biological functions in plants [1]. The data presented here revealed that disruption of *ASMT* caused a 2-fold decrease in the MT content and concomitant changes in the expression patterns of numerous genes. The classification of the DEGs identified by RNA-seq by their functions with respect to their role in MT effects suggests that these genes were primarily involved in hormone-mediated stress responses, which is consistent with the results of the in silico analysis of the *ASMT* promoter.

Putative cis-elements identified in *ASMT* promoter were associated, among other things, with the responses to ABA, ethylene, brassinosteroids, auxin, and methyl jasmonate. However, evaluation of *ASMT* promoter activity and qRT–PCR analysis of *ASMT* expression in response to hormone treatment did not always correspond to the expected results. The observed discrepancy is likely explained by the fact that some hormonal responses may be near saturation at a certain stage of development, especially in a wild type background reducing sensitivity of the experiment or that corresponding CREs were located in distal parts of the promoter outside the 1000 bp fragment. Modulations due to tissue-specific expression are also possible.

Our transcriptomic data of the *asmt* mutant demonstrated that enriched GO terms included terms related to auxin metabolism and signalling. which were downregulated in the mutant. Among them were numerous *SAUR* genes involved in plant cell expansion, shade avoidance, tropic growth (directional growth in response to environmental stimuli such as light and gravity), apical hook development, leaf growth, and senescence, suggesting the retardation of growth-promoting processes. In contrast to the general trend of decreased *SAUR* expression, the expression of *UGT74E2* (*AT1G05680*), which is related to auxin homeostasis, or *UGT73B4* (*AT2G15490*) was upregulated. These genes encode a UDP-glucosyltransferase that can transfer glycosyl groups to several compounds. Notably, in addition to transcriptional regulation, the amount of phytohormones can be regulated by UGT-mediated glycosylation, and the balance between a usually inactive glycoside and the corresponding active aglycone allows plants to accurately respond to environmental changes [27,28].

The intricate interplay between MT and other hormones also includes the ABA and ethylene pathways. ABA, which is well known for its universal stress-responsive behavior, generally interacts with MT through a negative cross-talk mechanism, although positive feedback interactions have been described [12,29]. The reduced expression of *NCED3*, encoding the rate-limiting enzyme for ABA biosynthesis, in the *asmt* mutant can be considered an example of positive regulation, demonstrating a highly variable mode of MT-ABA interaction. In parallel, the decreased expression of the highly ABA-inducible PP2C2 gene *HAI2* (*AT1G07430*), which has been shown to inhibit ABA signalling and activate gibberellic acid signalling, likely reflects compensation for the reduction in ABA content by enhancing ABA signalling [14].

ABA is coupled with ethylene production at various stages of plant growth and development and in defence responses. In *Arabidopsis*, ABA negatively regulates ethylene synthesis through ABI4-mediated transcriptional repression of the ethylene biosynthesis genes *ACS4* and *ACS8* [30]. Therefore, it is not surprising that under conditions of reduced expression of the ABA biosynthesis gene *NCED3*, the expression of ACS8 increased in the mutant. However, it cannot be ruled out that *ACS8* may play a dual role and may also participate in cysteine and methionine metabolism (ath00270 according to the KEGG pathway classification). As a result, *asmt* displayed a dramatically decreased expression of *MRD1* (*mto1 responding down*, *AT1G53480*), which was shown to be downregulated in the *mto1-1* mutant with the overaccumulation of soluble methionine [31]. To add the complexity, ACS8 is also essential for the defence response and early ethylene biosynthesis induced by copper [32].

Altered basal transcript levels of hormone-related genes contributed to the partially altered response of the mutant to hormone application as follows from the PCR results. Compared with those of the wild type, the responses of some genes were attenuated or even reversed, such as the expression of *PR1* induced by salicylic acid and *RAP2.6* treated with ABA or MeJa, indicating that the sensitivity of the mutant to hormonal stimuli was altered. The induction of *SAUR21* (*AT5G18030*) expression by IAA in the mutant and suppression in the WT plants is of particular interest since the auxin and SA signalling pathways are considered to be mutually antagonistic because of the negative effect of SA on auxin signalling [33]. Hence, the upregulation of some MT target auxin signalling genes in *asmt* may result from limitations in SA-mediated responses. In addition to their functions in plant defence responses to pathogenic infection, PR genes are strongly upregulated by abiotic stresses, including drought. In this context, the altered *PR1* expression in *asmt* could be interpreted as a display of impaired adaptive function, given that the mutant was enriched in GO terms related to water deficit.

Among the genes associated with water management, we identified DEGs encoding late embryogenesis abundant (LEA) proteins, which are required for membrane stabilization and enzyme protection. In addition to their role as protectants of biomolecules and membranes [34], LEA proteins are believed to function by enhancing photosynthetic efficiency and reducing ROS levels [35]. Compared with those in the WT, the steady-state expression levels of some *LEA* genes in the *asmt* mutant decreased, as well as their activation under water deficiency, in accordance with the drought-sensitive phenotype of the mutant. Notably, among the 51 *LEA* genes, only 7, encoding predominantly protein families 1 and 4 in the PFAM classification on the basis of primary sequences [36], responded to MT deficiency, which implies selectivity of MT-related regulation. The majority of the MT-regulated LEA proteins displayed dual localization in the cytosol and nucleus according to the subcellular distribution of the LEA proteome determined by Candat et al. [37], with the exception of vLEA30-RFP, which was among the MT targets and localized to the endoplasmic reticulum. This distribution is consistent with the central position of the cytosol, which interacts physically and functionally with all organelles and implies specific requirements of cellular compartments for stress protection.

Recently, a loss-of-function mutation of *PMTR1*, a putative MT receptor, has been shown to increase drought sensitivity in maize [38] and in *Arabidopsis* [39]. In particular, interactions between components of the MT signalling pathway CAND2 coupled with the G-protein α-subunit (GPA1) and the hydrogen peroxide sensor receptor kinase HPCA1 modulated stomatal closure and drought tolerance in *Arabidopsis.* Interestingly, most LEA genes whose expression decreased to steady state levels in the *asmt* mutant were expressed in a similar fashion in the *cand2* mutant impaired for the putative MT receptor PMTR1 (unpublished data). Therefore, both impaired synthesis and signalling may reduce drought tolerance, at least in part, by decreasing the ability of MT-targeted LEA proteins to protect biomolecules and membranes from desiccation or reactive oxygen species. This mechanism of MT activity contributes to the beneficial effects of MT under drought, which have been linked to its ability to regulate carbon and nitrogen metabolism [40] and maintain lower osmotic potential, facilitating higher turgor potential and relative water content [41].

Another potential molecular mechanism through which MT may increase drought tolerance involves ubiquitination, a posttranslational modification of proteins mediated by a cascade of ubiquitin ligases. MT deficiency in the *asmt* mutant contributed to more than a 2-fold downregulation of *AtAIRP1*, which encodes a cytosolic C3H2C3-type RING protein with E3 Ub-ligase activity. AtAIRP1 is involved in ABA-promoted stomatal closure and ROS production and acts as a positive regulator of the ABA-dependent response to drought stress, high salinity and low temperature in *Arabidopsis* [16]. Among the groups of RING domain-containing E3 ligases (SDIR1, AtAIRP1, AtAIRP2, AtAIRP3, AtAIRP4, RHA2a, and RHA2b), AtAIRP1 responded uniquely to MT deficiency. Given that AtAIRP1 is predicted to ubiquitinate negative regulators of components of ABA-dependent and/or signalling pathways, modulation of *AtAIRP1* by MT may allow plants to fine-tune their response to abiotic stresses, enhancing their adaptive capacity.

Impaired *ASMT* is functionally coupled with increased ROS production and reduced antioxidant capacity. Notably, the transcript abundance of some ROS-responsive genes, such as cold-sensitive *FH3* (*AT4G15200*) and *AT3G62960*, encoding thioredoxin superfamily proteins, was elevated in the *asmt* mutant compared with the wild type, even in the absence of stress. Similar patterns of increased expression of ROS-responsive genes were revealed by Li and Back in the *snat1* mutant defective in MT synthesis, whereas their induction under high light stress was significantly diminished [42]. The authors explain the increased steady-state expression of ROS-responsive genes by abnormal work of the ROS-scavenging system in part due to a lack of MT synthesis. It is conceivable that the elevated steady-state expression of stress-related genes in both cases is a result of adaptation to the stress state experienced by MT-deficient mutants under normal conditions.

Conclusively, our findings indicate that defects in MT synthesis induced by ASMT disruption affect other hormonal networks, presumably altering the hormonal balance and response to developmental and environmental cues. Furthermore, the discovery of new potential MT targets has contributed to the identification of specific mechanisms of MT activity through the modulation of the expression of genes involved in ubiquitination, chromatin folding, or RNA editing.

However, while recognizing the merits of mutants with impaired functions, such as *asmt* plants, it is worth noting that results obtained by means of reverse genetics should be treated with certain caution, since the observed effects may not be due to a direct disruption of the function of the gene being studied, but to the emergence of compensatory pathways for implementation of this function.

## 4. Materials and Methods

### 4.1. Plant Material, Growth Conditions and Treatments

*Arabidopsis thaliana*, ecotype Columbia-0, was used as a wild type plant. The T-DNA insertion mutant *asmt* (NASC680911) was obtained from the Nottingham Arabidopsis Stock Centre (Nottingham, UK) and confirmed by PCR analysis (Figure S1). The seeds were surface sterilized and stratified for 3 days in the dark at 4 °C on Petri dishes containing half-strength Murashige and Skoog (MS) medium supplemented with 1% sucrose and 0.5% agar. The plants were grown in a controlled growth chamber under a 16 h light/8 h dark photoperiod at 23 °C and 60 μmol m^−2^ s^−1^ irradiance for the indicated period.

For transcriptome analysis, 2-week-old plants were used. For the GUS experiments, 8-day-old p1000 *ASMT::GUS* seedlings were sprayed with half MS supplemented with corresponding phytohormones (5 × 10^−5^ M ABA, 5 × 10^−6^ M tZ, 10^−5^ M ACC, 10^−5^ M SA, 10^−7^ M EBL, 10^−6^ GA3, 10^−6^ M IAA, or 5 × 10^−5^ M MeJa) for 24 h. Hormonal concentrations and treatment times were selected for preliminary experiments. The effectiveness of treatment was confirmed via an expression analysis of marker genes specific for each hormone.

To study the response to water deficit induced by PEG treatment, two-week-old in vitro-grown seedlings were transferred to liquid MS media supplemented with PEG-8000 (25% [*w*/*v*]) for 4 h. For the dehydration experiments on adult plants, two-week-old plants were transplanted into soil and grown for another week. Three-week-old plants were subjected to drought stress by withholding water for the indicated periods.

### 4.2. Hormone Sensitivity Assays

To analyse sensitivity to ABA during the seed germination tests, the seeds were subsequently sown on MS media supplemented with 0.5 μM or 1 μM ABA. The germination rate was assessed by the number of seedlings exhibiting green and fully expanded cotyledons 6 days after sowing.

Hypocotyl measurements were performed with the seedlings grown in the dark for 4 days on MS plates supplemented with phytohormones at varying concentrations. Measurements were carried out in triplicate with 20 seedlings for each experiment.

For the chlorophyll retention assay, the 5th and 6th leaves were detached and incubated in the dark at 23 °C in water or hormone solutions for 3 to 6 days. The chlorophyll content (chlorophyll *a* + chlorophyll *b*) was determined as described by Lichtenthaler [43] and was related to the leaf area (mg/cm^2^). Three samples, each containing 3 leaf discs, were measured for each test. The chlorophyll content at the start of the experiment was taken as a reference and set at 100%.

### 4.3. Stress Tolerance Tests

Measurements of the relative water content of leaves (RWC) were performed on the basis of the methods of Nishiyama et al. [26]. After the initial determination of fresh weight (W), individual detached aerial parts of the stressed plants were placed in flasks and hydrated for 12 h in 40 mL of deionized water. The samples were then weighed (turgid weight, TW) and dried for 24 h to a constant weight (DW). The RWC was calculated as follows: RWC(%) = (W − DW)/(TW − DW) × 100.

Electrolyte leakage was measured with a Seven2Go S3 instrument equipped with an InLab 731-ISM electrode (Mettler Toledo, Columbia, MD, USA) as described by Nishiyama et al. [26]. Detached aerial parts of stressed plants (n = 5) were placed individually in flasks containing 40 mL of deionized water and shaken for 3 h. The percentage of electrolyte leakage was calculated as the percentage of the conductivity before boiling and after boiling.

The level of the TBARs (secondary products of the lipid peroxidation of membranes that react with thiobarbituric acid) was measured as described by Heath and Packer [44]. The measurements were carried out on a spectrophotometer (Ultrospec 2000; Pharmacia Biotech, London, UK).

The free proline content was estimated according to the methods of Bates et al. [45]. Fresh plant tissue (100 mg) was ground in liquid nitrogen, transferred to a plastic tube, added with 1 mL of distilled water, and incubated at 100 °C for 30 min. After centrifugation for 15 min at 13,000× *g* at 4 °C, the aqueous phase was collected and added with 1 mL of acetic acid and 1 mL of ninhydrin reagent (30 mL of glacial acetic acid + 20 mL of 6 M orthophosphoric acid + 1.25 g of ninhydrin). The mixture was incubated for 1 h at 100 °C and then cooled on ice. The absorbance of the solution was measured with a Genesys 10 UV spectrophotometer (Thermo Electron Corporation, Waltam, MA, USA) at 520 nm. The proline content was determined using a calibration curve and expressed in mM g^−1^ (FM).

The total antioxidant activity was evaluated via a DPPH assay adapted from Brand-Williams et al. [46]. The plant material was homogenized at 2–4 °C in 80% ethyl alcohol solution and centrifuged for 10 min at 8000× *g* at 4 °C. The reaction was initiated by adding 2 mL of 200 μM DPPH solution to 1 mL of the extract. The reaction mixture was incubated for 30 min at room temperature in the dark. The absorbance was measured at 517 nm.

The content of hydrogen peroxide in plant tissues was estimated by reaction with titanium chloride using the method of Kumar and Knowles [47].

The activity of superoxide dismutase (SOD) was assessed by reaction with nitroblue tetrazolium (NBT) according to the methods of Giannopolitis and Ries [48]. The activity of guaiacol-dependent peroxidase was determined according to the methods of Shevyakova et al. [49]. The reaction mixture contained 1 part supernatant, 20 parts 0.066 M potassium phosphate buffer (pH 7.4), 4 parts 7 mM guaiacol, and 4 parts μL 0.01 M H_2_O_2_. The absorbance of the solution was measured at 470 nm [49].

### 4.4. MT Measurement

A total of 0.1 g of plant tissue was ground in 1 mL of chloroform. The samples were centrifuged at 13,000 rpm for 10 min. The supernatant was collected in a clean tube. The samples were then placed in a centrifuge with a vacuum pump and dried for 2 h until all liquid evaporated. In total, 1 mL of distilled water was added to the sediment, and the samples were placed on a shaker for 3 h.

The endogenous MT concentration was detected with a quantitative sandwich enzyme-linked immunosorbent assay (ELISA) kit CEA908GE (Cloud-Clone Corp., Katy, TX, USA) according to the manufacturer’s protocol. The optical density was measured at 450 nm with a Multiskan MS LabSystems 352 microplate reader (Thermo/LabSystems, Pittsburgh, PA, USA).

### 4.5. Generation of ASMT pro::GUS-Transformed Arabidopsis Plants and Analysis of GUS Activity

#### 4.5.1. Construction of the ASMT Promoter::GUS Reporter Gene Fusion and Agrobacterium Transformation

*Arabidopsis* genomic DNA was extracted using the CTAB method. A 1000 bp region (−1000/−1) upstream of the translation start codon of *ASMT* was amplified from the genomic DNA via polymerase chain reaction (PCR) using forward and reverse primers carrying the BamHI and PstI restriction sites, respectively. The primers used for plasmid construction and sequencing are listed in Table S4. The resulting BamHI/PstI fragment was cloned upstream of the promoterless *uidA* reporter gene (*GUS*) of the binary vector *pCambia-1381Z* (Clontech, Mountain View, CA, USA), yielding the pCambia *ASMT pro*::*GUS* construct. After sequencing to exclude possible point mutations, the resulting construct was introduced into *Agrobacterium tumefaciens* strain GV3101 (C58) (GoldBio, St Louis, MO, USA) using the freeze–thaw method [50]. Positively transformed *A. tumefaciens* cells resistant to kanamycin (50 µg/µL) and rifampicin (100 µg/µL) were selected on LB plates at 28 °C for two days.

*Arabidopsis* plants in the Col-0 background were transformed with *A. tumefaciens* cells harboring P1000 plasmids using the floral dip method in the presence of 5% sucrose and 0.02% (*v*/*v*) Silwet L-77, as previously described by Clough and Bent [51]. The original genetic constructs of *pCambia1381Z* were used to generate negative and positive control plants, respectively, on the basis of GUS activity. T1 transgenic seeds from each transformed plant were tested for germination on half MS medium containing 0.8% (*w*/*v*) phyto agar (Duchefa Biochemie, Haarlem, The Netherlands) and 50 mg/L hygromycin (Hyg). Hyg-resistant seedlings were selected according to Harrison et al. [52] and then grown in soil at 22 °C under long-day conditions for further analysis. To prove the integration of the P1000 construct into Col-0 plants, total DNA was extracted from the rosette leaves of transgenic T1 plants and used for PCR analysis with *GUS*-specific primers (Table S4). For the GUS activity assays, three transgenic T2 lines with relatively high GUS gene expression were selected, and a representative line is shown.

#### 4.5.2. Histochemical Staining of GUS

Histochemical localization of β-glucuronidase activity in tissues was performed using the X-Gluc substrate, the hydrolysis of which yields an insoluble and highly colored indigo dye. Eight-day-old p1000 ASMT::GUS seedlings were sprayed with a solution of half-strength MS and phytohormones and incubated for 24 h. Staining was performed as described previously [53]. The results were recorded photographically using an MSP-2 stereomicroscope (LOMO, St. Petersburg, Russia).

#### 4.5.3. Fluorometric Assay of GUS Activity

GUS activity in solution was measured using the fluorometric substrate 4-methylumbelliferyl-β-D-glucuronide (4-MUG), which is hydrolysed to the fluorescent product 4-methylumbelliferone (MU). Approximately 50–100 mg of tissue was homogenized in 500 µL of extraction buffer (50 mM Na-phosphate buffer (pH 7.0), 10 mM EDTA, 10 mM β-mercaptoethanol, 0.1% sodium lauryl sarcosine, 0.1% Triton X-100) in a 1.5 mL centrifuge tube and then centrifuged for 10 min at 4 °C at 16,000 *g*. Fifty microliters of the supernatant was mixed with 500 µL of reaction buffer (extraction buffer + 0.5 mM 4-MUG) and warmed at 37 °C for 1 h in the dark. One hundred microliter aliquots were removed into labelled 1.5 mL centrifuge tubes containing 0.9 mL of stop buffer (0.2 M Na_2_CO_3_) to stop the reaction. The fluorescence was measured on a Hoefer DyNA Quant 200 fluorometer with emission and excitation filters set at 465 nm and 360 nm, respectively. A standard curve of fluorescence against the MU solution as a standard was plotted. The fluorescence was calculated as nmol MU/min/mg protein. The total protein concentration was measured by a BCA protein kit (Thermo Scientific, Waltham, MA, USA).

### 4.6. RNA Extraction and qRT-PCR

The frozen leaf samples were ground into powder in liquid nitrogen, and total RNA was extracted with TRIzol (Thermo Fisher, MA, USA) according to the manufacturer’s instructions. RNA was reverse transcribed using a mixture of oligo (dT) primers and random hexamers. The primer sequences for qRT-PCR were designed by Primer-BLAST (GenBank, NCBI, https://www.ncbi.nlm.nih.gov/tools/primer-blast/ accessed on 22 June 2026) and are presented in Table S4. qRT-PCRs were run on a LightCycler 96 (Roche, Switzerland) with hot start SYBR Green I technology. The PCR conditions were as follows: preheating at 95 °C for 5 min and 40 cycles of 95 °C for 15 s, 58 °C for 15 s and 72 °C for 25 s. The relative transcript abundance of the tested genes was calculated via the 2^−ΔΔCt^ method and normalized to the transcript level of the nuclear-encoded polyubiquitin *UBQ10* gene, which was used as an internal standard.

### 4.7. RNA Sequencing (RNA-Seq)

Total RNA was extracted from 2-week-old plants using a HiPure Total RNA Kit (Magen, Guangzhou, China). RNA concentration and purity were determined using a Bioanalyzer 2100 analyser (Agilent, Santa Clara, CA, USA). A total of 700 ng of total RNA with a RIN ≥ 7 was used for library construction via the NEBNext^®^ Poly(A) mRNA Magnetic Isolation Module and the NEBNext^®^ Ultra II™ Targeted RNA Library Prep Kit for Illumina (New England Biolabs, Ipswich, MA, USA) according to the manufacturer’s instructions. Paired-end sequencing was performed on a NovaSeq 6000 instrument (Illumina, San Diego, CA, USA). The raw sequences were transformed into clean reads with the parameters “ILLUMINACLIP::2:30:10 LEADING:20 TRAILING:20 SLIDINGWINDOW:4:15 MINLEN:30” in paired-end mode after data processing via Trimmomatic v. 0.36 [54]. The resulting high-quality clean reads were mapped to the reference genome of *A. thaliana* (version TAIR10.1), and reads per gene were counted via STAR v. 2.7.10b [55] with the parameters “—sjdbGTFfeatureExon exon —sjdbGTFtagExonParentTranscript transcript_id —sjdbGTFtagExonParentGene gene_id —quantMode GeneCounts”.

Identification and analysis of DEGs were performed via the “DESeq2” 1.48.0 R package [56] with thresholds of adjusted *p* value (FDR) < 0.05 and fold change > 2. The functional enrichment analysis included GO and KEGG term enrichment. Overrepresented DEGs were analysed via the Database for Annotation, Visualization, and Integrated Discovery (DAVID) online tool [57,58]. FDR-corrected *p* values < 0.05 and fold enrichment > 2 were used as thresholds to identify significantly enriched terms.

### 4.8. Statistical Data Analyses

Statistical analyses of gene expression data were performed with ANOVA with post hoc Holm multiple-comparison calculations using an online calculator (https://astatsa.com/OneWay_Anova_with_TukeyHSD/, accessed on 22 June 2026) and Student’s *t* test. All the data are presented as the mean values ± standard errors (SEs).

## 5. Conclusions

The results presented here demonstrate that *ASMT* gene expression is regulated by a number of hormones, among which ABA, ethylene, brassinosteroids, gibberellins and auxin play predominant roles, ensuring the participation of the enzyme in both developmental and protective processes. Limitations of MT metabolism promoted by *asmt* knockout mutation affect hormonal interplay, modifying the expression of genes involved in synthesis, signal transduction genes and responses to hormones. As a component of the stress response network, ASMT regulates the expression of target genes encoding specific LEA proteins involved in drought protection. Other targets that are highly dependent on ABA and may form ASMT functional modules associated with water deprivation include genes encoding PPR proteins, histones and ubiquitin ligases. These data suggest that in addition to the well-established role of MT as a ROS scavenger, this regulator may also trigger specific protective molecular mechanisms related to ubiquitination, chromatin folding, or RNA editing.

## Figures and Tables

**Table 1:** Physiological indices associated with antioxidant system activity in the wild type and *asmt* mutant. The data presented are the mean values ± SEs (*n* ≥ 3). Different letters denote statistically significant differences at *p* < 0.05 between the control and treatment (*t* test). Asterisks indicate statistically significant differences at *p* < 0.05 between the wild type and mutant (*t* test).

Treatment	WT	*asmt*
Total antioxidant capacity, relative units		
Mock	17.5 ± 0.89 ^b^	15.3 ± 1.13 ^b^
50 µM MT	20.1 ± 0.71 ^a^	26.8 ± 2.517 ^a^*
H_2_ O_2_, mM g^−1^ (FM)		
Mock	0.613 ± 0.025 ^a^	0.669 ± 0.020 ^a^*
50 µM MT	0.511 ± 0.019 ^b^	0.606 ± 0.017 ^b^*
SOD activity, relative units		
Mock	0.154 ± 0.009 ^a^	0.176 ± 0.008 ^a^*
50 µM MT	0.145 ± 0.008 ^a^	0.159 ± 0.007 ^b^
POD activity, relative units		
Mock	0.141 ± 0.013 ^a^	0.168 ± 0.012 ^a^
50 µM MT	0.144 ± 0.011 ^a^	0.152 ± 0.013 ^a^
Proline content, mM g^−1^ (FM)		
Mock	0.732 ± 0.022 ^a^	0.588 ± 0.019 ^a^*
50 µM MT	0.642 ± 0.012 ^b^	0.604 ± 0.012 ^a^*
TBARs, µM g^−1^ (FM)		
Mock	2.851 ± 0.185 ^a^	2.704 ± 0.096 ^a^
50 µM MT	2.871 ± 0.243 ^a^	2.604 ± 0.129 ^a^
Electrolyte leakage, percentage		
Mock	20.4 ± 1.1 ^a^	20.7 ± 1.6 ^a^
50 µM MT	21.1 ± 2.0 ^a^	20.2 ± 3.3 ^a^

**Table 2:** Expression of hormone-related genes (qRT-PCR) in the wild type and *asmt* mutant upon treatment with different hormones. The data presented are the mean values ± SEs (*n* ≥ 3). Different letters denote statistically significant differences at *p* < 0.05 between the control and treatment groups (ANOVA with post hoc Tukey’s multiple-comparison test or *t* test). Asterisks indicate statistically significant differences at *p* < 0.05 between the wild type and mutant (*t* test).

Treatment	WT	*asmt*
*NCED3*, transcript level (relative units)		
Mock	1.000 ± 0.058 ^a^	0.436 ± 0.031 ^a^*
50 µM ABA	0.219 ± 0.015 ^b^	0.218 ± 0.019 ^b^
*ACS8*, transcript level (relative units)		
Mock	1.000 ± 0.034 ^a^	2.338 ± 0.159 ^a^*
10 µM ACC	0.788 ± 0.054 ^a^	0.566 ± 0.035 ^b^
*PR1*, transcript level (relative units)		
Mock	1.000 ± 0.084 ^b^	0.523 ± 0.049 ^b^*
10 µM SA	11.880 ± 1.571 ^a^	3.142 ± 0.552 ^a^*
*NATA1*, transcript level (relative units)		
Mock	1.000 ± 0.099 ^b^	0.631 ± 0.059 ^b^*
1 µM IAA	254.200 ± 22.010 ^a^	528.700 ± 55.140 ^a^*
*RAP2.6*, transcript level (relative units)		
Mock	1.000 ± 0.055 ^c^	1.182 ± 0.101 ^c^*
50 µM ABA	13.902 ± 1.095 ^b^	10.110 ± 0.574 ^b^
50 µM MeJa	26.480 ± 1.675 ^a^	17.010 ± 1.467 ^a^*
*SAUR21*, transcript level (relative units)		
Mock	1.000 ± 0.112 ^a^	0.498 ± 0.043 ^b^*
50 µM MeJa	0.909 ± 0.067 ^a^	1.041 ± 0.092 ^a^
*ERF14*, transcript level (relative units)		
Mock	1.000 ± 0.035 ^a^	0.307 ± 0.021 ^b^*
10 µM ACC	0.750 ± 0.033 ^b^	0.663 ± 0.066 ^a^
*HAI2*, transcript level (relative units)		
Mock	1.000 ± 0.094 ^a^	0.494 ± 0.045 ^b^*
1 µM GA3	0.709 ± 0.069 ^b^	1.283 ± 0.115 ^a^*

## Data Availability

The original contributions presented in this study are included in the article/Supplementary Material. Further inquiries can be directed to the corresponding author.

## Supplementary Materials

The following supporting information can be downloaded at: https://www.mdpi.com/article/10.3390/ijms27135737/s1.
