# Supplementary material for: Loss of ASMT Function in Arabidopsis Affects Hormone Pathways and the Ability to Withstand Drought Stress
Source: Int J Mol Sci. 2026 Jun 25;27(13):5737. doi: 10.3390/ijms27135737 (PMC13362429; doi:10.3390/ijms27135737)
Supplement: Supplementary file 1 [file ijms-27-05737-s001.zip › ijms-4398127-supplementary.pdf]

Loss of ASMT function in Arabidopsis affects hormone pathways and the ability to withstand drought stress

Victoria V. Shitikova[1], Ivan A. Bychkov[1], Anna V. Klepikova[2], Anna S. Lifanova[1], Natalia V. Kudryakova [1]*, Elena S. Pojidaeva[1], Victor V. Kusnetsov[1]

[1] K.A. Timiryazev Institute of Plant Physiology Russian Academy of Sciences, Moscow, Russia
[2] N.I.Vavilov Institute of General Genetics Russian Academy of Sciences, Moscow, Russia
* Author for correspondence: nvkudryakova@mail.ru

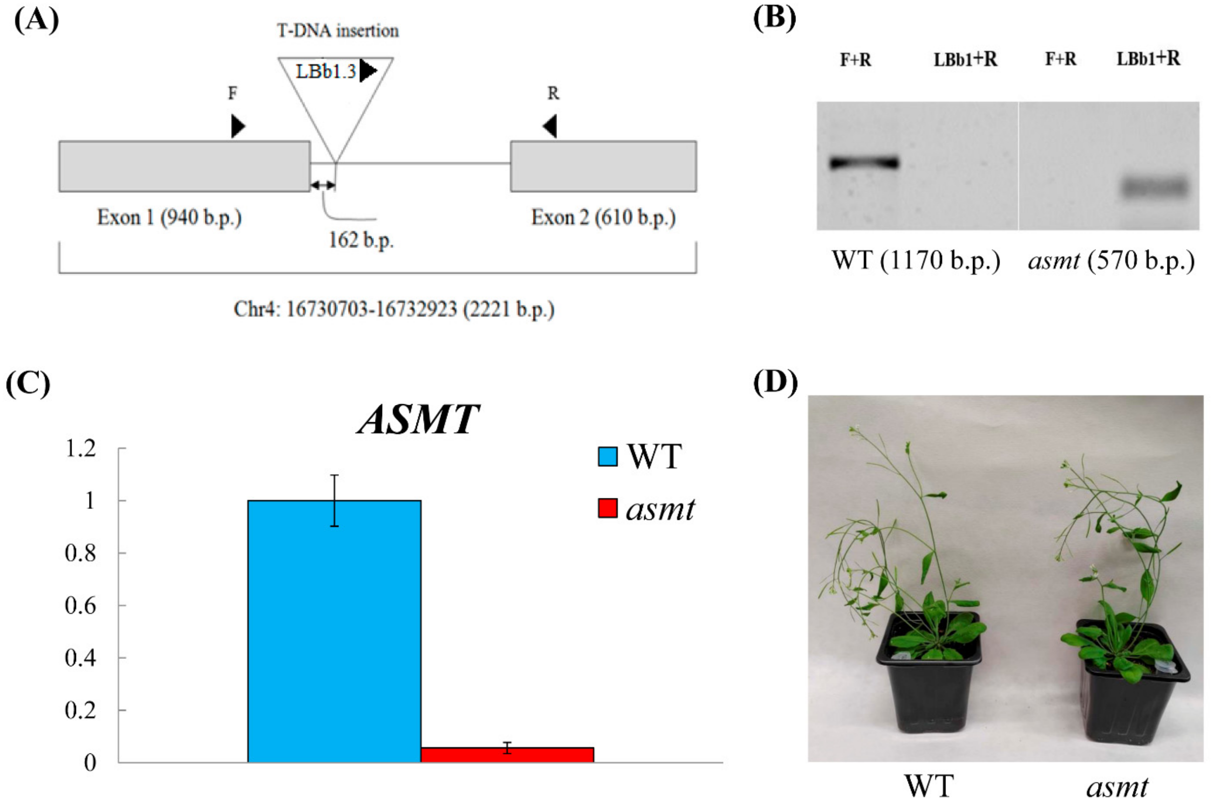

Figure S1. Characteristics of the *asmt* mutant. (A) Insertion position. (B) Genotyping results for the mutant line SALK_058444. (C) Transcript levels (qRT- PCR). (D) Phenotype of five-week-old wild-type and *asmt* mutant plants grown under 100 µmol m^{-2} s^{-1} light, 23 °C, and 16/8 h photoperiod.

Table S1. Differentially expressed genes (DEGs) in the *asmt* mutant.

Downregulated in the *asmt* against Col0

Locus	Official Symbol	Official Full Name	*asmt* vs Col0 log2 (expression ratio)
AT1G07430	*HAI2*	highly ABA-induced PP2C protein 2	-1,88
AT1G18265		zein-binding protein (Protein of unknown function, DUF593)	-1,18
AT1G20190	*EXPA11*	expansin 11	-1,08

AT1G31173		MIR167d	-1,09
AT1G32560	*AtLEA4-1*	Late embryogenesis abundant protein, group 1 protein	-1,77
AT1G34760	*GRF11*	general regulatory factor 11	-1,15
AT1G49500		transcription initiation factor TFIID subunit 1b-like protein	-1,25
AT1G52040	*MBP1*	myrosinase-binding protein 1	-1,76
AT1G52830	*IAA6*	indole-3-acetic acid 6	-1,28
AT1G52690	*LEA7*	Late embryogenesis abundant protein (LEA) family protein	-3,62
AT1G53480	*MRD1*	mto 1 responding down 1	-4,74
AT1G66100		Plant thionin	-1,26
AT1G74670	*GASA6*	Gibberellin-regulated family protein	-1,36
At2g03850		Late embryogenesis abundant protein (LEA) family protein	-2,90
AT2G15020		uncharacterized protein	-1,09
AT2G18050	*HIS1-3*	histone H1-3	-1,11
AT2G14610	*PR1*	pathogenesis-related protein 1	-1,87
AT2G21490	*LEA*	dehydrin LEA	-3,03
At2g23110		Late embryogenesis abundant protein, group 6	-2,08
AT2G26695		Ran BP2/NZF zinc finger-like superfamily protein	-1,13
AT2G32290	*BAM6*	beta-amylase 6	-1,07
AT2G32870		TRAF-like family protein	-1,55
AT2G33230	*YUC7*	YUCCA 7	-3,31
AT2G33830		Dormancy/auxin associated family protein	-1,02
AT2G40610	*EXPA8*	expansin A8	-1,21
AT3G02480		Late embryogenesis abundant protein, group 6	-2,77
AT3G03840	*SAUR27*	SAUR-like auxin-responsive protein family	-1,07
AT3G14440	*NCED3*	nine-cis-epoxycarotenoid dioxygenase 3	-0,95
AT3G17520		Late embryogenesis abundant protein (LEA) family protein	-3,21
AT3G18773		RING/U-box superfamily protein	-1,213
AT3G46900	*COPT2*	copper transporter 2	-1,14
AT3G53250		SAUR-like auxin-responsive protein family	-1,21
AT3G62550		Adenine nucleotide alpha hydrolases-like superfamily protein	-1,15
AT4G23450	*AIRP1*	RING/U-box superfamily protein	-1,16
AT4G29905		uncharacterized protein	-1,14
AT4G34550		F-box protein	-1,33
AT5G02020	*SIS*	E3 ubiquitin-protein ligase RLIM-like protein	-1,47
AT5G18030		SAUR-like auxin-responsive protein family	-1,31
AT5G18060	*SAUR23*	SAUR-like auxin-responsive protein family	-1,46
AT5G18080	*SAUR24*	SAUR-like auxin-responsive protein family	-0,99
AT5G39520		hypothetical protein (DUF1997)	-2,14
AT5G46871		Putative membrane lipoprotein (defensin-like family protein)	-1,02
AT5G54585		uncharacterized protein	-1,59

Upregulated in the *asmt* against Col0

Locus	Official Symbol	Official Full Name	*asmt* vs Col0 log2 (expression ratio)
AT1G03780	*TPX2*	targeting protein for XKLP2	0,99
AT1G04370	*ERF14*	Ethylene-responsive element binding factor 14	0,96
AT1G05680	*UGT74E2*	UDP-glucosyltransferase	1,01
AT1G34355	*PS1*	forkhead-associated (FHA) domain-containing protein	1,14
AT1G50240	*FU*	kinase family with ARM repeat domain-containing protein	0,98
AT1G62500		Bifunctional inhibitor/lipid-transfer protein/seed storage 2S albumin superfamily protein	1,07
AT2G04040	*DTX1*	MATE efflux family protein	0,97
AT2G15490	*UGT73B4*	UDP-glycosyltransferase 73B4	0,97
AT2G18193		P-loop containing nucleoside triphosphate hydrolases superfamily protein	1,00
AT2G36780		UDP-Glycosyltransferase superfamily protein	1,25
AT2G39030	*NATA1*	Acyl-CoA N-acyltransferases (NAT) superfamily protein	1,13
AT3G62960		Thioredoxin superfamily protein	1,21
AT4G15200	*FH3*	formin 3	1,30
AT4G17030	*EXLB1*	expansin-like B1	1,03
AT4G24110		NADP-specific glutamate dehydrogenase	1,28
AT4G29030		Putative membrane lipoprotein	1,09
AT4G35180	*LHT7*	LYS/HIS transporter 7	1,85
AT4G37370	*CYP81D8*	cytochrome P450, family 81, subfamily D, polypeptide 8	1,16
AT4G37770	*ACS8*	1-amino-cyclopropane-1-carboxylate synthase 8	1,43
AT5G08490	*SLG1*	Tetratricopeptide repeat (TPR)-like superfamily protein	1,18
AT5G22140		FAD/NAD(P)-binding oxidoreductase family protein	1,55
AT5G25260		SPFH/Band 7/PHB domain-containing membrane-associated protein family	1,11

Table S2. List of *cis*-regulatory elements identified in the *ASMT* gene promoter by the AGRIS database.

Motifs	Position on chromosome (start)	Position on chromosome (end)	Conservative motive (5’->3’)	Family	Function

ABRE-like binding site motif	16734549	16734556	*tacgtgta*		ABA Responsive Element. The ABRE-like motif 5'-*ACGT*-3' is part of several overlapping motifs, including motifs for binding basic leucine zipper proteins (bZIPs), which are often transcriptional activators. Together with other motifs, they may be involved in the dehydration response, including during seed maturation and germination, etc.
ARF1 binding site motif	16734611	16734616	*tgtctc*	ARF	Auxin-sensitive elements
	16734853	16734858			
	16733250	16733255			
	16733952	16733957			
	16733250	16733255			
	16734853	16734858			
	16734611	16734616			
	16733952	16733957			
ATB2/AtbZIP53/AtbZIP44/GBF5 BS in ProDH	16734715	16734720	*actcat*	bZIP	A TF-sensitive motif inducible under hypoosmolar conditions.
AtMYC2 BS in RD22	16734603	16734608	*cacatg*	bHLH	An ABA-responsive sequence was found in the ABA-inducible gene *RD22*. This sequence is involved in ABA stimulation and binds to the drought-inducible MYC homolog. Response to ABA (induction), response to MYC. Drought-sensitivity element.
	16734542	16734547			
Bellringer/replumless/pennywise BS1 IN AG	16733084	16733091	*aaattaaa*	Homeobox x	Multidirectional regulation of gene expression depending on tissue specificity
	16733833	16733840			
CCA1 binding site motif	16734018	16734025	*aaaaatct*	MYB-related	CCA1 is a Myb-like transcription factor, CIRCADIAN CLOCK ASSOCIATED1 (CCA1), capable of initiating and establishing the phase of circadian clock-controlled rhythms.
DPBF1&2 binding site motif	16734291	16734297	*acactag*	bZIP	Motifs targeting the DPBF (Dc3 promoter binding factor, a bZIP-class transcription factor), which shares up to 96% DNA binding similarity with ABI5. This motif is characteristic of genes expressed in seeds.
	16734040	16734046	*acacacg*		
	16733976	16733982	*acacgag*		
	16733357	16733363	*acaccag*		
	16734542	16734548	*acacatg*		
	16733316	16733322	*acaccag*		
	16734602	16734608	*acacatg*		
GATA promoter motif [LRE]	16733813	16733818	*tgataa*		Light-sensitive elements

	16732814	16732819	*agatag*		
	16732972	16732977	*tgataa*		
	16733046	16733051	*agataa*		
	16734234	16734239	*agatag*		
	16734506	16734511	*tgataa*		
	16735081	16735086	*agatag*		
MYB1 binding site motif	16733411	16733418	*atccaacc*	MYB	Response to various physiological reactions
	16733195	16733201	*accaaac*		
	16734282	16734288	*aacaacc*		
	16735053	16735059	*aactaac*		
	16734643	16734649	*accaaac*		
	16733195	16733202	*aaccaaac*	MYB	
	16734643	16734650			
RAV1-A binding site motif	16733511	16733515	*caaca*	ABI3VP1	RAV1-A and RAV1-B are *cis*-binding elements that mediate the binding of ABA-dependent transcriptional activators of the ABI3/VP1 family.
	16734269	16734273			
	16735179	16735183			
	16733206	16733210			
	16733078	16733082			
	16735066	16735071	*cacctg*		
SORLREP3	16734973	16734981	*tgtatatat*		Light-sensitive elements
T-box promoter motif	16734448	16734453	*actttg*		Light-sensitive elements
	16733394	16733399			
	16734805	16734810			
W-box promoter motif	16733056	16733061	*ttgact*		Response to various physiological processes, including defense against pathogens, aging, and development. They can act as both transcriptional activators and repressors.
	16733592	16733597			
	16733455	16733460	*ttgacc*		
	16732821	16732826	*ttgact*		

Table S3. Expression of hormone marker genes in wild type and mutant *asmt*.

Treatment	WT	*asmt*
ARR5, transcript level (relative units)		
Mock	1.000±0.075[b]	1.526±0.089[b*]
5 µM tZ	18.830±1.262[a]	20.000±1.615[a]
DWF4, transcript level (relative units)		
Mock	1.000±0.071[a]	2.129±0.214[a*]
100 nM EBL	0.528±0.076[b]	0.832±0.049[b]
IAA19, transcript level (relative units)		
Mock	1.000±0.118[b]	1.267±0.111[b]
1 µM IAA	4.616±0.802[a]	3.175±0.268[a*]
PR1, transcript level (relative units)		

Mock	1.000±0.084[b]	0.523±0.049[b*]
10 µM SA	11.880±1.571[a]	3.142±0.552[a*]
RD29, transcript level (relative units)		
Mock	1.000±0.191[b]	1.055±0.155[b]
50 µM ABA	168.100±1.570[a]	184.600±0.552[a]
ERF1, transcript level (relative units)		
Mock	1.000±0.227[b]	0.714±0.127[b*]
10 µM ACC	1.853±0.315[a]	1.439±0.231[a]
GA3, transcript level (relative units)		
Mock	1.000±0.099[a]	0.859±0.059[a]
1 µM GA3	0.461±0.041[b]	0.294±0.070[b*]
PDF1.2, transcript level (relative units)		
Mock	1.000±0.030[b]	0.690±0.050[b*]
50 µM MeJa	11.350±1.210[a]	17.710±1.7204[a*]

Table S4. List of primers used for RT-qPCR.

Locus	Description	Forward 5'-3'	Reverse 5'-3'
AT1G32070	*SNAT1*	TACGCAACTTGTGGAACCACCT	ATCATAAACATCAATCTCACCACCA
AT1G26220	*SNAT2*	GATGGAGCGGTTGATTGAGG	GTCAGAGACGAACCCAAGCG
AT4G35160	*ASMT*	GCAAAGAAGCGGTCCCTCCAA	GTCCGTTCTTTGCCTGTGCTTGT
AT5G54160	*COMT*	GGAGTGACGAACATTGCGT	TCTCGGTTCGTTCTTTGCCT
AT3G60290	*M2H*	AGGTTCTTTGACTTACCTGCCG	GTAGCAGGGAGGATTGGAAGG
AT1G17020	*M3H*	CAGGAAAGAAGCCGACATGGA	TAGGAGGAACGGTCGTGATCG
AT3G05010	*CAND2*	ATGCGAGTGCTCAGCGAGAT	CGACCCATCCGCCGATAAA
AT2G26300	*GPA1*	CGTTTGCGAGTGGTTCAGAGAT	CCAAAGCCGTCGTCCTGTAGAT
AT1G43160	*RAP2.6*	GTTGGAACTCAGACGATTCAACG	CCTACTAATGGTTGTTGTTGCTCCA
AT5G01830	*SAUR21*	ATCCGCTCATCTCTGCTTCG	AGGTCAAAGTCATGGAGCGG
AT3G14440	*NCED3*	GCTGCGGTTTCTGGGAGAT	GGCGGGAGAGTTTGATGATT
AT4G37770	*ACS8*	GGTTTTCCGGCTATCGTTTCA	CACACTGCATTATCCGTTACA

AT1G04370	*ERF14*	GGATCAAGGAGGTCGTAGCAGTGG	TTATTGCCTCTTGCCCATGTTG
AT1G07430	*HAI2*	CGGACTGTGACGCTGTTGGAT	CACTCGTCCTCCTGCTTCTTGG
AT2G39030	*NATA 1*	CAGTGAAGTTGGGTGTCGGAAG	CATCGCCAGTGAGCCTACAAAG
AT3G48100	*ARR5*	TATCTACTCGCAGCTAAAACGC	GTAAGCCGAAAGAATCAGGACA T
AT3G50660	*DWF4*	GTTGGCCATTTCTTGGTGAAA	TGGCGGTGTACGGTTTAAGAT
AT3G15540	*IAA19*	ACGAAGACAAAGATGGAGACTGGA	GTCATCATCACTCGTCTACTCCT CT
AT2G14610	*PR1*	TTCACAACCAGGCACGAGGA	GCAGCGTAGTTGTAGTTAGCCTT C
AT3G23240	*ERF1*	ATTAGGGTTTGGCTCGGGAC	GACTCTTGAACTCTCTCCGCCG
AT1G15550	*GA3*	AGTCTCTTCGGGCTACCTGT	CGGAAATCGTTGAGAGGCG
AT5G44420	*PDF1.2*	TGTTCTCTTTGCTGCTTTCGACG	GCATGATCCATGTTTGGCTCCT
AT1G52690	*LEA7*	GTCGGAAACTGGAGAAGCAATCA	GCCATCTGTTTCACCTGCTCAC
AT3G17520	*LEA30*	CAACATCCAACCCACTCACACC	GATTTAGCTGTCTCGTATGCCGA A
AT3G02480	*LEA28*	CTGCTTCAGCTCAAGACTCCTTG	GTCTTGTCCTTGACGACATCAGC
AT5G66400	*RAB18*	TCGCATTCGGTCGTTGTATTGT	AGTAAACAACACACATCGCAGG A
At4G05320	*UBQ10*	GCGTCTTCGTGGTGGTTTCTAA	GAAAGAGATAACAGGAAC GGAAACA
PCR from plasmid pCambia-1381Z	*GUS*	TGATTGATGAAACTGCTGCTGCTG	GCTTGCTGAGTTTCCCCGTTG
